# Beyond the Pleura: A Case Report of Type 1 Brugada Phenocopy in the Setting of Empyema

**DOI:** 10.7759/cureus.90135

**Published:** 2025-08-15

**Authors:** Jia Wei Tan, Nashriq Khan Adam Khan, Akmal Hallman, Nur Edriana Hizreen Mohd Hizam

**Affiliations:** 1 Endocrinology and Diabetes, Norfolk and Norwich University Hospital, Norwich, GBR; 2 Acute Medicine, Norfolk and Norwich University Hospital, Norwich, GBR

**Keywords:** brugada phenocopy, brugada sydrome, chest infection, pleural empyema, right-sided pleural effusion

## Abstract

We report a case of a 50-year-old male with a history of Hodgkin lymphoma in the neck region (in remission), ischaemic heart disease, and hypothyroidism who presented with right-sided pleuritic chest pain, cough, fever and night sweats. Imaging revealed a large right-sided loculated pleural effusion. Electrocardiogram (ECG) demonstrated a spontaneous Type 1 Brugada pattern, absent in prior recordings. Investigations ruled out intracardiac masses and the patient was treated for empyema with antibiotics and chest drainage, leading to clinical improvement. A follow-up ECG after four weeks of oral antibiotics was performed and revealed resolution of the Type 1 Brugada ECG pattern. This strongly suggested that the Type 1 Brugada ECG pattern was associated with the empyema, especially in the absence of family history or structural heart disease. This case highlights the diagnostic challenge of distinguishing Brugada syndrome from Brugada phenocopy (BrP), particularly when an infectious trigger is involved. Empyema has not been widely reported as a cause of BrP, making this a unique clinical observation. Thorough evaluation, serial ECGs, and consideration of reversible causes are essential to avoid unnecessary interventions such as implantable cardioverter-defibrillator (ICD) implantation. Right-sided empyema may represent a rare but reversible cause of Brugada phenocopy. Awareness of such presentations can aid accurate diagnosis and prevent misclassification of Brugada syndrome in acutely unwell patients.

## Introduction

Brugada syndrome (BrS), first described in 1992 by Josep and Pedro Brugada, is an autosomal dominant genetic disease with incomplete penetrance associated with an increased risk of sudden cardiac death (SCD) [1]. Type 1 Brugada syndrome is identified by the coved ST segment elevation >2 mm in more than one of V1-V3 followed by a negative T wave, whereas Type 2 is characterised by >2 mm of saddleback shaped ST elevation, and Type 3 can have similar morphology to either Type 1 or Type 2, but with <2 mm of ST segment elevation. Brugada syndrome affects approximately three to five individuals per 10,000 and is significantly more common in males. It is commonly diagnosed in adulthood average age of around 40 years, though it has occasionally been reported in children. It is primarily caused by the mutation of the SCN5A gene, which causes a loss of function of the sodium channel, which in turn impairs the fast upstroke in phase 0 of the action potential and leads to slow conduction in the heart [2].

It is important to be aware of Brugada phenocopy (BrP), which manifests as transient Brugada-like ECG patterns seen in the absence of true BrS. The phenocopy can be caused by identifiable and reversible conditions such as metabolic disturbances, myocardial ischemia, mechanical compression, or drug effects. The distinction between the two is therefore of critical clinical importance: misdiagnosing BrP as BrS could lead to unnecessary interventions such as implantable cardioverter-defibrillator (ICD) placement, while failure to identify true BrS could result in missed opportunities to prevent fatal arrhythmic events. Accurate diagnosis ensures that patients receive appropriate risk stratification, management, and, when indicated, genetic counselling and family screening.

## Case presentation

We present a case of a 50-year-old man with a history of Hodgkin’s lymphoma in the neck region in remission for 20 years, ischaemic heart disease with angioplasty two decades ago, and hypothyroidism presented with Type 1 Brugada electrocardiogram (ECG) changes in the setting of right-sided lung empyema. The patient presented with a two-week history of pleuritic right-sided chest pain worsened with movement and deep inspiration and was associated with productive cough (green sputum), night sweats, and abdominal bloating. He did not report weight loss, lumps or bumps in the body, or other constitutional symptoms. Prior to presentation, he had completed two antibiotic courses (doxycycline and amoxicillin) with minimal improvement. He is an active smoker (10-15 cigarettes/day) of 30 years and consumes alcohol occasionally. He admitted to poor adherence to his levothyroxine medication, often missing doses for one to two weeks at a time.

Physical examination revealed stony dullness on percussion up to the middle zone of the right hemithorax, with reduced breath sounds and voluntary guarding in the right upper quadrant, no cardiovascular abnormalities were noted. Laboratory investigations showed significantly raised inflammatory markers as shown in Table 1.

**Table 1:** Laboratory investigations showing elevated inflammatory markers indicative of an infective process.

Test	Result	Unit	Reference Range
Total White Cell (TWC)	31.8	×10 /L	4.0 – 10.0 ×10 /L
Platelet Count	69	×10 /L	150 – 410 ×10 /L
C-reactive Protein (CRP)	314	mg/L	0 – 5 mg/L
Ferritin	1185	µg/L	23 – 300 µg/L

**Figure 1:**
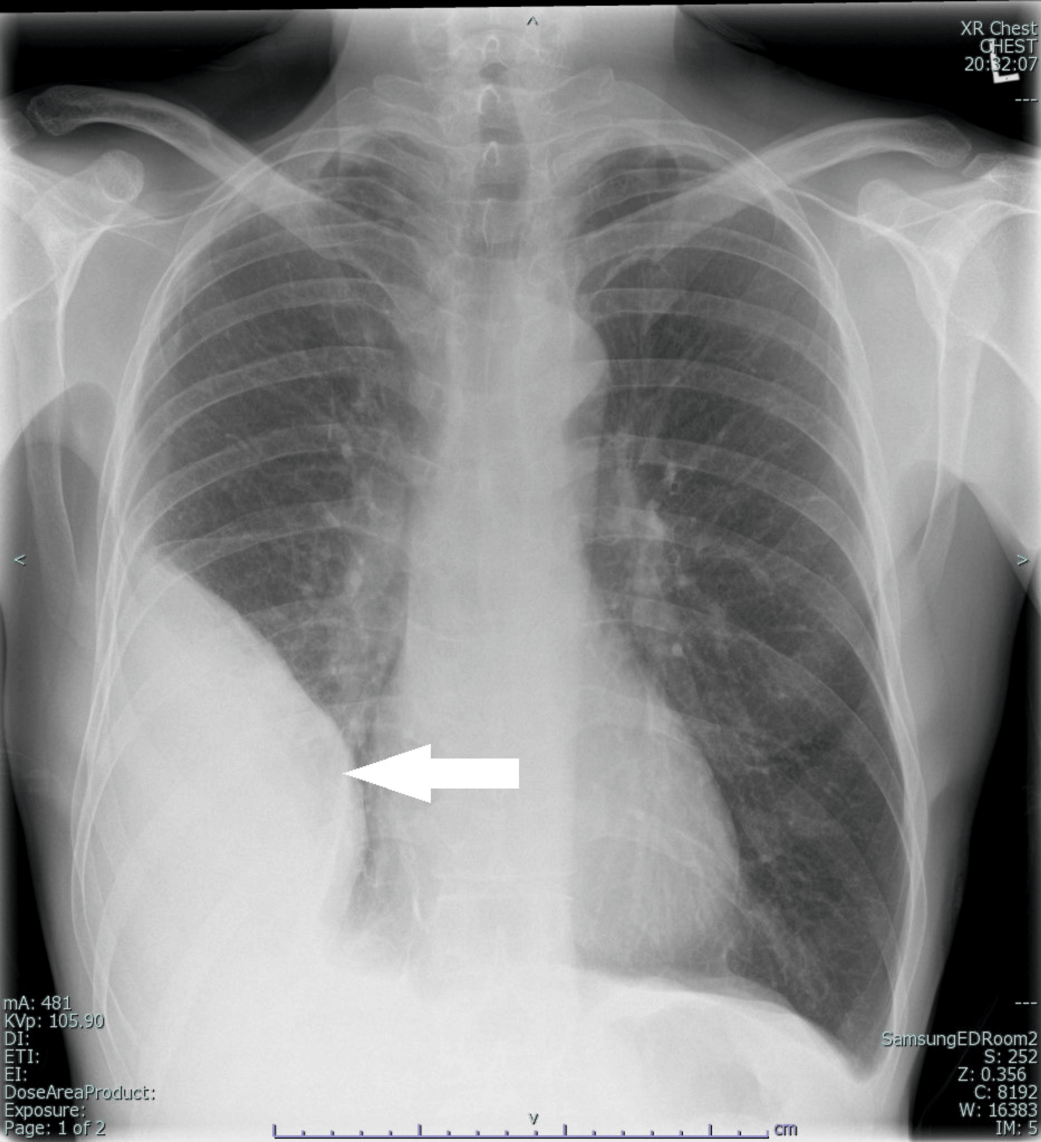
Arrow in chest X-Ray shows a large subpulmonic collection within the right mid to lower zone.

**Figure 2:**
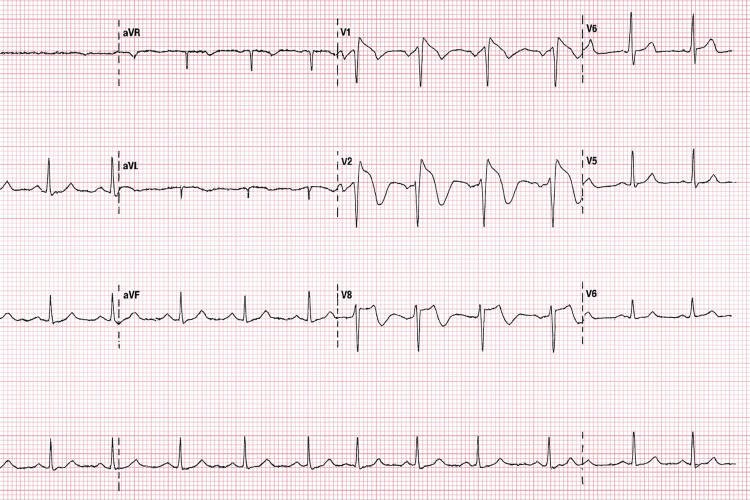
ECG on admission that that shows Type 1 Brugada ECG pattern with coved ST segment elevation followed by a negative T wave in V1-V3 ECG - Electrocardiogram

**Figure 3:**
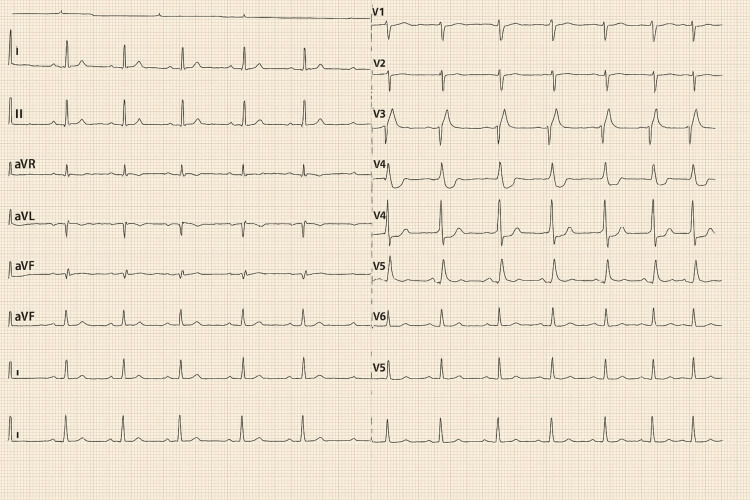
Previous ECG in 2002 that shows sinus rhythm with no Type 1 Brugada ECG pattern ECG - Electrocardiogram

Chest radiograph demonstrated a large right pleural effusion (Figure 1). ECG performed showed a Type I Brugada pattern (Figure 2), a new finding compared to a prior ECG from 2002 during the admission for treatment of previous Hodgkin lymphoma (Figure 3).

**Figure 4:**
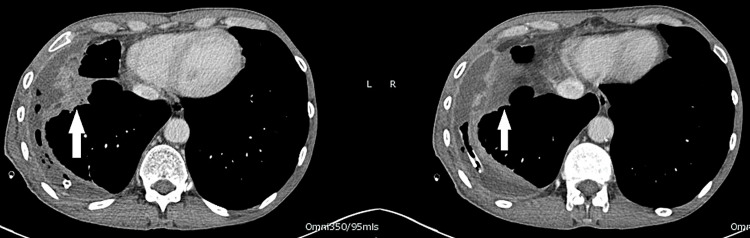
CT Thorax images showing moderate right pleural collection containing fluid and locules of gas (arrow) CT: Computed tomography

The patient was co-managed by Cardiology and Respiratory teams. An urgent transthoracic echocardiogram (TTE) was performed to look for possible intracardiac mass given his history of lymphoma. This showed normal left ventricular systolic function, mild aortic and mitral regurgitation, with no evidence of cardiac mass or external compression. The respiratory team diagnosed a right-sided empyema and treated the patient with intravenous piperacillin/tazobactam and doxycycline. A right intercostal chest drain was inserted. Pleural fluid culture grew Streptococcus intermedius, sensitive to penicillin. Cytology confirmed empyema with no malignant cells. A contrast-enhanced computed tomography (CT) of the thorax, abdomen, and pelvis confirmed right-sided empyema with consolidation of the right middle and lower lobes (Figure 4).

After five days of intravenous antibiotics and chest drainage, the patient improved clinically. Repeat chest radiograph showed partial resolution. He was prescribed oral co-amoxiclav for 35 days with outpatient respiratory follow-up.

**Figure 5:**
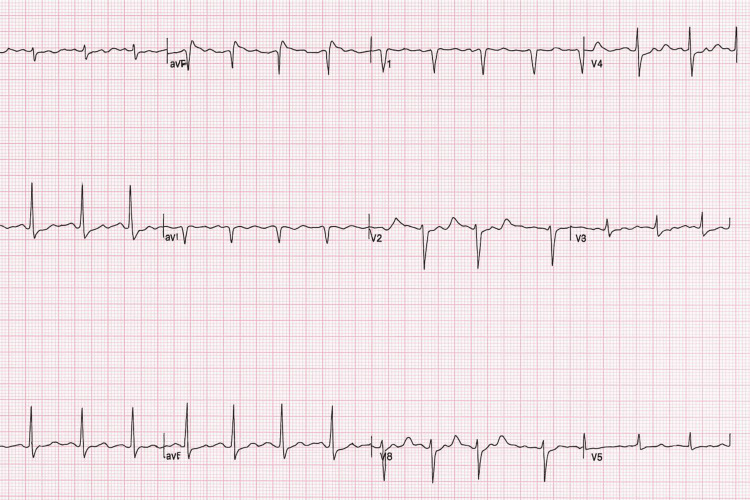
ECG showing resolution of Type 1 Brugada pattern following antibiotics back to sinus rhythm ECG - Electrocardiogram Notice the ECG resolution of coved ST segment elevation and T wave inversion in this figure back to sinus rhythm compared to the ECG in Figure 2.

The cardiology team recommended that the ECG should be repeated to assess for persistence of the Brugada pattern after treatment of the lung infection. If the pattern remained, genetic testing for Brugada syndrome was planned. Patient was subsequently reviewed again in the respiratory clinic with a follow-up CT thorax after four weeks of oral co-amoxiclav. Although the patient was clinically well, the CT Thorax revealed an increase in size of the right pleural empyema. Owing to concerns regarding potential deterioration of the empyema if the antibiotics were withdrawn, the patient was referred to the thoracic surgery team. Here, left video-assisted thoracoscopic surgery (VATS) with left anterolateral thoracotomy was conducted with further samples obtained with microbiology and histology laboratory analysis. An ECG recorded during this thoracic surgical admission showed that Brugada-type ECG findings had resolved back to sinus rhythm, as shown in Figure 5.

## Discussion

Suspicion of Brugada syndrome arises when an individual presents with characteristic ECG findings and a suggestive family history of SCD. Given the high risk of ventricular tachyarrhythmias and SCD associated with the syndrome, recognising this distinctive ECG pattern is crucial. However, it is also important to distinguish between Brugada syndrome and Brugada phenocopy. Riera et al. introduced the term phenocopy, which describes “an environmental condition that imitates one produced by a gene” and this serves as a reasonable and succinct description for all acquired Brugada-like ECG manifestations [3]. In other words, the term ‘Brugada phenocopy’ refers to patients who exhibit Brugada-like ECG patterns without having the genetic or clinical substrate of true Brugada syndrome.

In Brugada syndrome, the ST-segment elevation is caused by a conduction delay in the right ventricular outflow tract (RVOT). The delayed depolarisation of the RVOT creates an abnormal current, which, in turn, leads to ventricular arrhythmia [4]. At a cellular level, in Brugada phenocopy, the ST-segment elevation can be explained by a transmural gradient resulting from an accentuated Ito‐mediated action potential (AP) notch and a loss of the AP dome in the epicardium, but not in the endocardium [5]. Baranchuk et al. claimed that Brugada phenocopies may result from the cardiac sodium channel blocking effects of structural abnormalities in the heart, particularly those affecting the right ventricle [5].

The diagnosis of BrP relies on six criteria, with the first four being mandatory. These are: i) characteristic Type I or Type II Brugada ECG pattern, ii) the presence of an underlying condition inducing Brugada ECG patterns, iii) resolution of the ECG patterns after eliminating the underlying condition, iv) a low pretest probability for BrS as defined by lack of clinical symptoms, medical history and family history suggestive of BrS, v) negative provocative testing with sodium channel blocker drugs (not mandatory if surgical RVOT manipulation has occurred within the last 96 hours), and vi) a negative genetic testing for SCN5A (recommended but not mandatory as it is possible to identify mutation only in 20%-30% of probands that are known to have BrS) [6]. This case is noteworthy for highlighting the diagnostic uncertainty between BrS and BrP in the setting of an acute infection - a rarely reported trigger for phenocopy.

As mentioned above, several triggers of Brugada phenocopy have been reported, including electrolyte imbalance, mechanical compression, myocardial ischaemia, pericardial disease, infiltrative cardiomyopathy and pulmonary embolism. A few hypotheses have been proposed regarding the triggers mentioned above. However, infection, especially empyema, has not been identified as a cause, although a few reports have documented Brugada phenocopy during COVID-19 infections [7], where the arrhythmia was induced by fever. There was also another case study involving a patient who had no history of major adverse cardiovascular events (MACE) and family history of SCD, presented with a type 1 Brugada pattern, believed to have been induced by a high fever secondary to pneumonia [8].

Our patient had a history of non-Hodgkin lymphoma diagnosed 20 years ago, which was in remission. Brugada phenocopy secondary to lymphoma has been reported in a few cases, either due to mediastinal tumours or cardiac lymphoma [9-11]. Therefore, when this patient initially presented with a Brugada-type ECG pattern, it was essential to rule out cardiac lymphoma or external cardiac compression, and based on the above investigations, fortunately, there was no evidence of malignancy or lymphoma recurrence. As the follow-up ECG showed resolution of the Brugada-type pattern after drainage of the empyema via intercostal chest drain, the Brugada type 1 pattern was likely attributable to the empyema, a finding that has not been previously reported.

Moreover, this patient had no signs or symptoms suggestive of Brugada syndrome and no family history of cardiac disease or SCD, making a diagnosis of Brugada syndrome very unlikely (low pre-test probability), though he was known to have ischaemic heart disease. Therefore, an ICD is not considered necessary in this case, as there is no evidence to support ICD implantation in this group of patients [12]. In such cases, genetic testing for the SCN5A gene may be considered, particularly if the Brugada-type ECG pattern persists following resolution of the empyema which was suggested by our cardiology team. 

One of the key learning points from this case is to highlight the importance of taking a thorough history and performing ECG, even in non-cardiac presentations - particularly in cases like this, where cardiac findings might easily be overlooked due to more obvious clinical features. Serial ECGs and outpatient follow-up should also be arranged to monitor the pattern and any subsequent resolution.

## Conclusions

This case highlights the importance of distinguishing Brugada syndrome from Brugada phenocopy, particularly in the setting of acute infection. While phenocopies have been linked to various reversible conditions, empyema remains an uncommon and under-recognized trigger. The resolution of the Brugada-type ECG pattern following treatment supports a diagnosis of phenocopy rather than true Brugada syndrome. Clinicians should maintain a high index of suspicion for reversible causes in patients presenting with Brugada-pattern ECGs and pursue further evaluation only if ECG changes persist after resolution of the underlying condition. Accurate diagnosis is essential to prevent unnecessary interventions such as ICD implantation.
